# Use of prone position in spontaneous breathing in patients with COVID-19

**DOI:** 10.2478/jccm-2025-0015

**Published:** 2025-04-30

**Authors:** Rodrigo Cerqueira Borges, Isadora Salvador Rocco, Camila Botana Alves Ferreira, Mauricio Kenzo Tobara, Cristiane Helena Papacidero, Vanessa Chaves Barreto Ferreira, Andrey Wirgues Sousa

**Affiliations:** Hospital Samaritano Higienópolis, São Paulo, Brazil; Universidade Federal de Sao Paulo, Brazil

**Keywords:** COVID-19, ICU, prone position

## Abstract

**Objective:**

To investigate if awake prone position (PP) reduces the rate of endotracheal intubation and mortality in patients with COVID-19 admitted to the intensive care unit (ICU).

**Methods:**

This was a retrospective cohort study of 726 patients who were admitted to the ICU with acute hypoxic respiratory failure secondary to COVID-19. The protocol of the institution recommended the use of awake PP in patients with nasal catheter with an oxygen flow ≥ 5 L/min and SpO_2_ ≤ 90% or a high-flow nasal catheter (HFNC) with FiO_2_ ≥ 50% and SpO_2_ ≤ 90%. The following data were collected: age, comorbidities, SAPS-3 score, onset of symptoms, the degree of pulmonary involvement, duration of invasive and noninvasive MV, HFNC therapy, nitric oxide therapy, hemodialysis and PP while spontaneously breathing.

**Results:**

There was a higher mortality rate in the supine position group (27.1%) than in the awake PP group (13.9%). There was no significant difference in the time on MV or number of patients on MV (p>0.05). The variables with p < 0.05 in the bivariate analysis were entered into the Cox regression model. The model was adjusted for awake PP, sex, age, SAPS-3 score, onset of symptoms, the degree of pulmonary involvement, chronic arterial disease, and noninvasive ventilation. The only variable associated with lower mortality over time was awake PP (hazard ratio: 0.55; 95% confidence interval: 0.33–0.92).

**Conclusion:**

Awake prone position has been shown to be a safe and effective therapy that reduced mortality but not the risk of intubation in patients with COVID-19.

## Introduction

COVID-19 is an acute respiratory syndrome caused by severe acute respiratory syndrome coronavirus 2 that was first described in the Wuhan region of China and has since become the most serious global health crisis of the last century. The disease resulted in a significant increase in critically ill patients requiring admission to the intensive care unit (ICU) due to severe hypoxemia [1]. In these patients, the incidence of acute respiratory distress syndrome (ARDS) is high, ranging from 33% to 68% [1,2,3]. Currently, one of the treatments for ARDS not related to COVID-19 [4] is positioning mechanically ventilated patients in the prone position, which has been shown to improve oxygenation and reduce mortality.

In recent years there has been great interest in the effects of the prone position in patients on admitted to the ICU for several reasons, such as reducing mortality, the favorable physiological benefits to oxygenation, increasing functional residual capacity, reducing dead space and intrapulmonary shunts, increasing ventilation in gravity-dependent areas, and relieving the weight that the heart exerts on the lungs [5,6]. All these benefits mentioned above have been demonstrated in patients on invasive mechanical ventilation. Thus, it has been speculated that these benefits of the prone position could also occur in non-mechanically ventilated patients with Covid-19. However, to date the physiological effects and clinical benefits of the awake prone position in these patients remain uncertain.

Some studies have used the awake prone position in patients with oxygen therapy or non-invasive ventilation and found a reduction in respiratory effort and hypoxemia in patients with Covid-19, which may be particularly beneficial given the increased risk of self-inflicted lung injury [7,8]. Thus, favoring the reduction of the intubation rate and improving survival in patients with Covid-19 [9,10,11]. Despite this, the uncertainties about the effectiveness of the awake prone position in COVID-19 are still substantial [12]. Therefore, the objective of this study was to evaluate the effects of the awake prone position on reducing the rate of endotracheal intubation and mortality in patients with COVID-19 admitted to the intensive care unit.

## Methods

This was an observational, retrospective cohort study with 726 patients admitted to three different ICUs of a single hospital with a diagnosis of COVID-19 between March 2020 and February 2022. The study protocol was approved by the ethics committee of the institution. Written informed consent was not considered necessary due to the retrospective design of the study. We included all ICU patients who were older than 18, who had a confirmed diagnosis of COVID-19 by real-time polymerase chain reaction test, who required oxygen supplementation (≥3 L/min), and who did not require invasive mechanical ventilation in the first 24 hours in the ICU. Patients were excluded if they had hemodynamic instability, were referred to another hospital, had incomplete clinical records (not enough information on whether the patient was treated in the prone position or no data on the main study outcomes), were readmitted to the ICU during the same hospital stay and were not considered eligible again, or had previous tracheostomy.

### Awake prone position

The protocol of our institution recommended that the prone position could be used in spontaneously breathing patients with a nasal catheter with a flow ≥5 L/min and SpO_2_ ≤90% or with a high-flow nasal catheter (HFNC) with FiO_2_ ≥50% and SpO_2_ ≤90% who showed no signs of hemodynamic instability. The benefits and risks of the prone position were explained to these patients, and if the patients agreed, they were encouraged to spend as much time as possible in the prone position, according to their tolerance, but not within 1 hour after meals to avoid gastrointestinal side effects. The prone position sessions were interrupted if oxygenation improved over the initial indication criteria or if the patient was discharged from the hospital, intubated, or died. In cases of worsening respiratory failure or clinical deterioration, the institution’s default criteria for endotracheal intubation were as follows: respiratory rate >35 breaths/minute, fatigue, respiratory acidosis with pH <7.25, copious tracheal secretions, severe hypoxemia with SpO_2_ <90%, FiO_2_ ≥0.6, hemodynamic instability, deterioration of mental status, or doctor’s decision. These criteria were followed to avoid late intubations.

Patients breathing spontaneously with noninvasive ventilation or oxygen who performed the prone position were called the awake prone position group and those who did not performed the supine position group.

### Variables analyzed

Data were collected from electronic medical records and were reviewed by physical therapists working in intensive care according to a previously standardized protocol. Patient confidentiality was protected by assigning a code to each (deidentified) patient. The following variables were collected: age, sex, comorbidities, SAPS-3 score, time of onset of symptoms, the degree of pulmonary involvement on CT, need for invasive mechanical ventilation, duration of invasive and noninvasive mechanical ventilation, need for high-flow therapy, duration of high-flow therapy, nitric oxide level, hemodialysis, time in the awake prone position, time in the prone position with invasive mechanical ventilation, and length of hospital stay.

### Statistical analysis

The sample size was calculated to detect a 10% difference in the incidence of intubation between the groups at α =95% and β =90%, based on the results of previous studies [11,12,13]. The calculated sample size was 618 patients, with 309 patients per group. A total of 25% more patients were added to the data collection, anticipating loss or incomplete completion of primary outcome data or transfers to other hospitals. The descriptive results are presented as mean ± standard deviation or median (interquartile range) for quantitative variables and as n (%) for qualitative variables. Student’s t test or the Mann‒Whitney U test was used, as appropriate, for the comparison of quantitative variables, and the chi-squared test was used for the comparison of qualitative variables. Differences in mortality rates between groups were assessed using Kaplan‒Meier curves. Significance was determined using the log-rank test. Cox proportional-hazards regressions were adjusted to assess the effect of awake prone position on mortality, adjusting for potential confounders. In parallel, an analysis will be made of the subgroup of patients who remained in the awake prone position for more than 4 hours compared to those who remained for less than 4 hours. The significance level was set at 5% (p<0.05), and the samples were analyzed with SPSS-17 statistical software (SPSS Inc., Chicago, IL, USA).

## Results

A total of 831 patients with a confirmed diagnosis of COVID-19 were admitted to our ICUs during the study period. Of them, 25 were transferred to another hospital, 70 received invasive mechanical ventilation within the first 24 hours of ICU admission, and 11 underwent tracheostomy on admission. Thus, 726 patients had full data for analysis and were included.

Table 1 describes the general characteristics of the patients admitted to the ICUs. A total of 289 patients were evaluated in the awake prone position group. Patients in the awake prone position group were 56.8 ± 13.7 years old and the supine position group 63.5 ± 17.4 years old (p<0.05). There were proportionally more male patients (69.2%) in the prone position group than in the supine position group (59.4%; p<0.05). There was no difference in the prevalence of comorbidities between the groups, except that chronic arterial disease was more prevalent in the supine position group (p<0.05). The SAPS-3 score was higher in the supine position group (52.0 (32.5–63.5) vs. 45.0 (29.0–53.0)). The proportion of patients with a PaO_2_/Fio_2_ ratio <200 in those who required mechanical ventilation was 85.1% in the awake PP group and 77.4% in the supine position group (p>0.05) throughout the hospitalization.

**Table 1.** General characteristics of patients admitted to the ICU with COVID-19

**Variables**	**Supine position (n=437)**	**Awake prone position (n=289)**	**p**
Age, years	63.5 ± 17.4	56.8 ± 13.7	0.001^*^

Gender, male, n (%)	259.0 (59.4)	200.0 (69.2)	0.007^*^

Comorbidities			
Diabetes Mellitus, n (%)	142.0 (32.6)	87.0 (30.1)	0.472
Systemic arterial hypertension, n (%)	218.0 (50.0)	129.0 (55.4)	0.157
Chronic heart failure, n (%)	28.0 (6.4)	15.0 (5.0)	0.238
Dyslipidemia, n (%)	94.0 (21.6)	52.0 (18.0)	0.241
COPD, n (%)	28.0 (6.4)	18.0 (6.2)	0.917
Chronic arterial disease, n (%)	35.0 (8.0)	11.0 (3.0)	0.020^*^
Obesity, n (%)	104.0 (23.9)	95.0 (32.9)	0.080
Onset of symptoms, days	7.0 (4.0–9.0)	8.0 (6.0–10.0)	0.001^*^
SAPS-3	52.0 (32.5–63.5)	45.0 (29.0–53.0)	0.001^*^

Involvement pulmonary on chest tomography			0.001^*^
0–25% changes, n (%)	150.0 (34.5)	51.0 (17.7)	
25–50% changes, n (%)	214.0 (49.2)	185.0 (64.2)	
>50% changes, n (%)	71.0 (16.3)	52.0 (18.1)	

Quantitative variables are presented as median (25–75th percentile), except in special conditions, which are presented as mean ± standard deviation. Qualitative data are presented as an absolute number (percentage). COPD= chronic obstructive pulmonary disease.

* p<0,05 comparison between supine position and awake prone position groups.

Among the variables analyzed in the ICUs, the proportion of patients who had noninvasive ventilation was greater in the prone position group (77.9% vs. 65.0%, p<0.05). The same was true for the proportion (p<0.05) and duration of high-flow therapy (p< 0.05). The number of patients on mechanical ventilation was similar between the groups (p>0.05), as was the total time on mechanical ventilation (p>0.05). There was no difference in cases of moderate and severe ARDS in the first 24 hours after intubation between the groups (p>0.05). The mortality rate was greater in the supine position group (27.1% vs. 13.9%) than in the awake PP group (p<0.05). These and other variables analyzed in the ICUs are described in Table 2. The time in the awake PP was 4 hours/day (IQR 2–8 hours/day), with a median of 2 days (IQR 1–4 days). No significant adverse events were reported, but the most common limitations were position intolerance (4%), back pain (4%), anxiety (3%), discomfort (2%), and loss of peripheral venous access (1%).

**Table 2.** Variables analyzed during the ICU stay in patients with COVID-19

**Variables**	**Supine position (n=437)**	**Awake prone position (n=289)**	**p**
Non-invasive ventilation, n (%)	284.0 (65.0)	225.0 (77.9)	0.001^*^
Non-invasive ventilation, days	2.0 (1.0–5.0)	3.0 (2.0–5.0)	0.008^*^
High flow therapy, n (%)	221.0 (50.6)	228.0 (78.9)	0.001^*^
High flow therapy, days	3.0 (2.0–6.0)	4.0 (2.0–6.0)	0.006^*^
Mechanical ventilation, n (%)	219.0 (50.1)	141.0 (48.8)	0.727
Mechanical ventilation, days	13.0 (7.0–23.5)	12.0 (7.0–28.0)	0.475
Moderate or severe ARDS in the first 24 hours after intubation (PaO_2_/FiO_2_ ratio <200)	122.0 (27.9)	82.0 (28.3)	0.896
Prone in Mechanical Ventilation, n (%)	88.0 (20.3)	52.0 (18.0)	0.655
Tracheostomized in the ICU, n (%)	69.0 (15.8)	46.0 (15.9)	0.963
Hemodialysis, n (%)	87.0 (19.9)	44.0 (15.2)	0.258
ICU time, days	20.0 (12.0–34.5)	17.0 (12.0–30.0)	0.120
Length of hospital stay, days	27.0 (12.0–36.0)	23.0 (11.50–33.5)	0.730
Mortality, n (%)	118.0 (27.1)	40.0 (13.9)	0.001^*^

Quantitative variables are presented as median (25–75 percentile). Qualitative data are presented as an absolute number (percentage).

* p<0,05 comparison between supine position and awake prone position groups.

**Fig.1.**
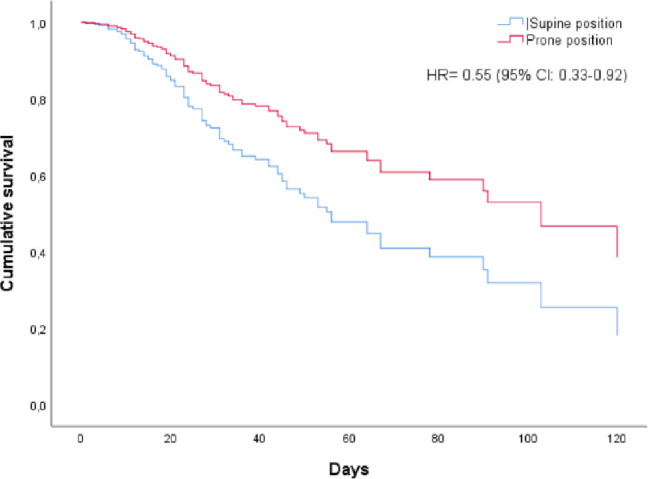
Mortality of the two groups over time

The Cox regression analysis for mortality is shown in Table 3. The only factor associated with the death rate was prone position while spontaneously breathing (hazard ratio 0.558; 95% confidence interval (CI) 0.338–0.921). Figure 1 shows the mortality of the two groups over time. Compared with supine position group, the awake PP reduced mortality over time (log-rank p<0.05). There was no difference in mortality rate or intubation rate between the subgroups stratified by time spent in the awake PP (more or less than 4 hours daily) (p>0.05).

**Table 3.** Cox regression analysis with factors associated with mortality in patients with covid 19

	**Exp (B)**	**Wald**	**p**	**95% CI for EXP (B)**	
Involvement pulmonary	1.299	1.295	0.255	0.828	2.040
SAPS-3	1.005	0.607	0.436	0.992	1.018
NIV time	0.988	0.620	0.431	0.958	1.019
Age	1.016	3.442	0.064	0.999	1.034
Gender	1.523	3.702	0.054	0.992	2.338
Time of onset of symptoms	1.008	0.146	0.703	0.969	1.048
Chronic arterial disease	0.966	0.009	0.925	0.469	1.991
Awake Prone position	0.558	5.216	0.022	0.338	0.921

## Discussion

In this observational study, the association between awake prone position and the rate of intubation and mortality in hypoxemic patients with Covid-19 was evaluated. The awake prone position was associated with a lower mortality rate than the supine position, having no effect on the rate of intubation. These results on the care of hypoxemic patients with COVID-19 reinforce the clinical benefits that this strategy has shown in patients with COVID-19 [11,14], suggesting that awake prone positioning may reduce mortality in these patients.

Unlike our study, a systematic review evaluating the effects of awake prone position on mortality found only a trend toward improvement, with no significant effect, over usual care (11% vs. 22%) [12]. This difference can be explained in part by the number of patients included in that systematic review. Thus, in a meta-analysis that included more patients (n=2352), the prone position was associated with a lower death rate (odds ratio 0.57, 95% CI 0.36–0.93; P=0.02) [14], like our result (hazard ratio 0.55, 95% CI 0.33–0.92; p=0.02).

The beneficial effect of the prone position on mortality can be explained by several factors. For example, the supine position alters lung function in patients with respiratory failure due to gravitational differences between dependent and nondependent regions, resulting in more negative pleural pressure, increasing transpulmonary pressure in nondependent areas (more distension), and producing the opposite effect in dependent areas where the pleural pressure is less negative and the transpulmonary pressure is lower (less distension). Ventilation in the prone position causes a uniform distribution of transpulmonary pressure, favoring uniform ventilation [4,11]. The prone position also increases oxygenation in patients with respiratory failure, especially by improving the ventilation/perfusion ratio [(4,11]. Thus, the physiological effects of the prone position are beneficial to oxygenation, making death less likely.

Patients in the awake prone position group were younger and had lower severity scores, which could explain this difference in mortality between the groups. On the other hand, they had greater degree of pulmonary involvement on CT, with 82% of the patients having more than 25% involvement on CT versus only 65% in the supine position group. Nevertheless, after adjustment for the regression analysis, only the prone position and severity score were associated with reduced mortality. To date, it is still not possible to predict who will benefit from the awake prone position. Weatherald et al [15] compared groups with different severities of hypoxemia (SpO_2_/FiO_2_<150 vs. SpO_2_/FiO_2_≥150) found a protective effect in those with more severe hypoxemia, who had a relative risk for intubation of 0.77 (0.64 to 0.92). This was also observed in those with a duration of therapy >5 hours (relative risk = 0.78 (0.66 to 0.93)) [15]. In contrast, we did not find a link between the severity of hypoxemia and the duration of therapy. Our data are in agreement with a recent meta-analysis that found no association between subgroups and prone position [16]. This study was not designed to evaluate patients who responded to the prone position, so further studies are needed to explore the effect of the prone position in different subgroups.

The prone position did not reduce the need for intubation in patients with COVID-19. The mechanism by which the prone position might reduce the need for endotracheal intubation remains unclear. A meta-trial of six randomized controlled trials [17], including selected patients with COVID-19 with a HFNC, reported a reduction in intubation rates up to day 28 of hospitalization. The intubation rates in that meta-trial were lower than those presented in this study (33% vs. 48%), which may reflect the differences in intubation practices and protocols established for the initiation of mechanical ventilation in our institution compared to those in the meta-study, which may have caused us to implement intubation earlier (i.e., more readily) and thus reduced the effects of the prone position on the likelihood of intubation. This idea may be reinforced by a recent meta-analysis [15], which concluded that the prone position could reduce the intubation rate in patients with COVID-19, with an absolute effect of 55 fewer intubations per 1,000 patients. Therefore, studies evaluating the minimum or maximum time to determine the improvement or failure of prone position are necessary to avoid early or late intubations.

In addition, it is important to understand when the prone position should be initiated or, specifically, at what stage (mild versus moderate hypoxemia). Tolerance is an important limitation, and it is not feasible to aim for a similar duration of the prone position in spontaneous breathing to that of intubated patients (>16 h/day). For example, the longest duration of the prone position achieved in observational studies of awake patients was 8 hours. In patients on mechanical ventilation [4], the effect of the prone position may depend on the length of time [18]. We found no difference in the intubation rate or mortality rate when comparing the >4-hour vs. <4-hour prone position group in spontaneously breathing patients. These results are similar to those observed by other authors, who did not find differences when analyzing the effects of 3.4 hours versus 9 hours of awake prone position on intubation and death [19]. It does seem well supported that protocols should target prone positioning for at least 1 hour, as this is associated with shorter hospital stays and intubation [17,20].

Some authors argue that the awake prone position may be a risk because it causes a transient improvement in oxygenation, leading to a false sense of safety and delaying the installation of invasive support [14]. This view stems from the improvement in oxygenation without reducing the vigorous spontaneous inspiratory efforts that can potentially aggravate lung damage, leading to patient self-inflicted lung injury (P-SILI) [21,22]. This concept has polarized different ideological positions, and for some it is a cause for concern to advocate that intubation and invasive degree of pulmonary involvement be started as soon as possible to prevent disease progression [23,24]. We disagree with this reasoning because we did not find negative outcomes for those who used awake prone position when a protocol for the installation of mechanical ventilation was used. In addition, no difference was found in mortality or duration of mechanical ventilation between critically ill patients with COVID-19-related respiratory failure who were intubated early and those who were intubated later [25]. Therefore, P-SILI should not be a justification for performing early intubation without indication, and the harm associated with elective early intubation may outweigh the theoretical benefit.

The SAPS-3 score was not associated with mortality in the regression model. COVID-19 is a disease with a unique clinical profile, with often unpredictable manifestations and disease progression, and rapid drastic changes in its clinical picture. Some characteristics of the disease, such as thrombosis and an exacerbated systemic inflammatory response, may not be fully captured by the SAPS-3, which was developed prior to this pandemic. Additionally, mortality from COVID-19 is influenced by a range of factors, including the availability of healthcare resources, therapeutic strategies adopted, and the characteristics of the circulating viral variant. SAPS-3 alone may not have been able to capture all these nuances. Additionally, some authors have reported that SAPS 3 should be used with caution in patients with Covid-19 [26].

The percentage of patients undergoing hemodialysis was not different between the groups. The combination of kidney disease associated with acute triggering events, dialysis complications, comorbidities and the need for intensive care makes dialysis patients potentially more severe compared to non-dialysis patients, which could influence the increase in mortality in this group of patients and generate a bias in the results.

The prone position for spontaneous breathing seems to be safe and has the advantage of allowing patients to interact with their families during hospitalization, favoring the humanization of care. Patient adherence is essential but is often influenced by factors such as back pain, intolerance, and anxiety, which increase the likelihood of refusal and nonadherence. Nevertheless, in the present study, the incidence of these events was low. This high adherence may have occurred because all patients were aware of the procedure, and the whole care team was trained in the prone protocol and prepared for practical considerations, such as the optimization of analgesia, adequate communication, and patient assistance to improve comfort, adherence, and proper positioning, especially during the initial sessions in a clinically deteriorating patient.

In bivariate comparisons, there was no association between comorbidities and the rate of mechanical ventilation in patients in prone or supine position. The relationship between comorbidities and the need for mechanical ventilation in patients with COVID-19 is complex and may vary in different cases. It is important to understand that the presence of comorbidities increases the risk of developing severe forms of the disease and, consequently, the need for mechanical ventilation. However, there are situations in which this relationship may not be so direct, for example, the severity of SARS-CoV-2 infection may be the determining factor for the need for mechanical ventilation, regardless of the number or type of comorbidities.

Study limitations: 1) The observational nature of the study may reduce the methodological quality needed to evaluate the effect of the intervention under these conditions. Nevertheless, many patients were evaluated, and the sample size had enough power to detect a significant result of the primary outcome of the study. 2) The number of hours in the prone position varied between patients according to their tolerance, which made it difficult to establish a minimum time necessary to obtain the clinical benefits of the intervention. 3) Though the variables that describe the severity of patients may be a source of bias, the regression analysis adjusted for confounding variables demonstrated the benefits of therapy on the outcomes mortality. 4) Due to the characteristics of the study, variables such as SpO_2_, PaO_2_/FiO_2_ or the ROX index were not collected before and after the prone sessions. Therefore, the individual responses could not be tracked, limiting our ability to analyze the effects of prone intubation in specific patient subpopulations. However, our data showed no effect of prone time on the risk of death or the need for intubation. 5) Patients were encouraged to perform the prone position only 1 hour after meals. This could reduce the time they spent daily in the position. However, we opted for this strategy because some patients reported abdominal discomfort when remaining in the position immediately after meals. We standardized this period in our protocol to still allow a large part of the day for therapy. 6) Another fact is that we could partially allow gastric emptying and the risk of bronchoaspiration. However, current evidence shows that a much longer period than the one we used would be necessary to reduce satisfactory emptying [27]. 7) The number of patients collected in the awake prone position group was close to the calculated sample number. It was not possible to reach the calculated sample number because there was a sharp decline in hospitalizations of patients due to Covid-19 until the expected end date of the study.

## Conclusion

The awake prone position in hypoxemic patients may be a safe and effective therapy that reduces mortality but not the risk of intubation in patients with COVID-19. The frequency, duration, and criteria for starting and interrupting intubation remain unknown.
